# Relief for Spasmodic Asthma

**Published:** 1866-11

**Authors:** 


### *Relief for Spasmodic Asthma.*

Dr. J. S. Monell, in a communication to the *Medical Record* on this subject, writes as follows:

"Having been a sufferer from frequent, severe, and protracted attacks of spasmodic asthma, for a period of fifteen years, and having, by accident, hit upon a means for speedy relief, I am induced to present the same to the profession, in the hope that by its adoption it may prove as beneficial to such as are subject to attacks of this distressing affection as it has been to myself and to some of my patients.

"In December, 1865, I was having a severe attack of asthma one evening about nine o'clock. I placed myself standing at the foot of my bed, with my arms folded on the foot-board for a pillow, the forehead resting on the folded arms, and the feet placed at a sufficient distance to make a partial semi-circle of the body. While laboring severely for air, the thought occurred to me to cease breathing for a few seconds. I did so, and after several trials I felt some relief. I then expired all the air that it was possible to, after which I determined not to inspire again until I found it absolutely necessary. I succeeded in waiting several seconds; then inspiration was carried to its fullest capacity, and retained with great effort for many seconds. This act of forced expiration, waiting, thorough inspiration, and again waiting, was continued for some fifteen minutes, and, to my delight, the spasm was perfectly relieved. I have since relieved several similar attacks, by the same method, in less than two minutes.

"I have advised this course for many others, and their testimony has been uniformly satisfactory, except in one instance, that of an aged lady with heart disease. It requires a great effort on the part of the patient to perform the act. It is well for the medical adviser to perform it personally in presence of the patient, and then desire him to perform it once or twice under his supervision. The first attempt of retaining the inspired air during the asthmatic attack will cause the patient to think he cannot continue it, but perseverance will soon delight him with relief from the spasm."

